# The Prevalence, Distribution, and Extent of Subclinical Atherosclerosis and Its Relation With Serum Uric Acid in Hypertension Population

**DOI:** 10.3389/fcvm.2021.638992

**Published:** 2021-04-15

**Authors:** Fei Liu, Simei Hui, Tesfaldet H. Hidru, Yinong Jiang, Ying Zhang, Yan Lu, Haichen Lv, Sharen Lee, Yunlong Xia, Xiaolei Yang

**Affiliations:** ^1^Department of Cardiology, First Affiliated Hospital of Dalian Medical University, Dalian, China; ^2^Faculty of Medicine, Chinese University of Hong Kong, Hong Kong, China

**Keywords:** hypertension, subclinical atherosclerosis risk factor, uric acid, cardiovascular disease, risk factor

## Abstract

**Background:** Data are limited on the prevalence, distribution, and extent of subclinical atherosclerosis (SCA) in populations with primary hypertension and an in-depth evaluation is required to explore the impact of elevated serum uric acid (SUA) levels on the systemic extent of SCA.

**Methods:** A total of 1,534 individuals with blood pressure-controlled primary hypertension registered from January 1, 2015 to May 31, 2018 were included. The systemic extent and risk factors of SCA in the carotid, coronary, thoracic, and renal territories were investigated by Doppler ultrasound and computed tomography.

**Results:** SCA was present in 85.9% of patients. The proportion of focal, intermediate and generalized SCA was 17.9, 21.3, and 46.6%. Plaques were most common in the thoracic aorta (74%), followed by the coronary (55.3%), carotid (51.6%), and renal (45.8%) arteries, respectively. Participants were stratified into quartiles based on gender-specific SUA levels. Compared with patients in the first quartile, the Odds Ratio (OR) [95% confidence interval] for SCA in the second, third and fourth quartile were 1.647 (1.011–2.680), 3.013 (1.770–5.124), and 5.081 (3.203–10.496), respectively. Patients with elevated SUA levels at high 10-year Framingham risk had a higher likelihood of a more severe risk of SCA (95.8%). However, extensive SCA was also present in a substantial number of low 10-year-Framingham risk patients at the higher quartiles of SUA (53.8%).

**Conclusions:** SCA was highly prevalent in the hypertension population and the thoracic aorta was the most frequently affected vascular site. Elevated SUA concentration was significantly associated with the prevalence and severity of SCA regardless of territories.

## Introduction

Subclinical atherosclerosis (SCA) refers to the existing arteriosclerosis with no significant clinical symptoms of severe atherosclerotic stenosis, or SCA in arteries [coronary artery, cerebral artery, renal artery, and peripheral artery (1)]. Published documents revealed that SCA is prevalent in people with traditional cardiovascular risk factors such as high blood pressure, dyslipidemia, obesity, diabetes, and smoking (2). However, currently there is only one published report to our knowledge that detailed the prevalence, vascular distribution, and multi-territorial extent of SCA among the general population (3). Hence, data is lacking among patients with hypertension.

Recent studies have found that high concentrations of serum uric acid (SUA) is associated with kidney, cardiovascular, cerebrovascular, and peripheral vascular target organ damages (4). Also, previous studies reported that high concentrations of SUA independently is associated with cardiovascular risk and events in both the general population and in patients with hypertension (5–7). However, other studies on uric acid and target organ damage reported no significant association between high levels of SUA and cardiovascular events (8, 9). Consequently, the association of high levels of SUA and SCA remains controversial and further studies are required.

The natural progress of atherosclerosis comprises a protracted subclinical phase, with the disease often diagnosed following a cardiovascular event or lately at an advanced stage (10). It is well-established that cardiovascular events are associated with poor health outcomes. Also, many deaths secondary to coronary artery disease occurs suddenly in patients with hypertension (11). Tangible scientific evidence on risk and identification of SCA is needed to improve the prevention and treatment of atherosclerotic diseases (12). In extension, it is of great significance to reduce the risk of cardiovascular events by strengthening the identification of patients with early atherosclerotic disease through non-invasive or minimally invasive methods. In this context, we sought to assess the prevalence, vascular distribution, and multi-territorial extent of SCA, and explore the relationship between SUA levels and SCA in hypertensive patients.

## Methods

### Study Sample

**Figure 1:**
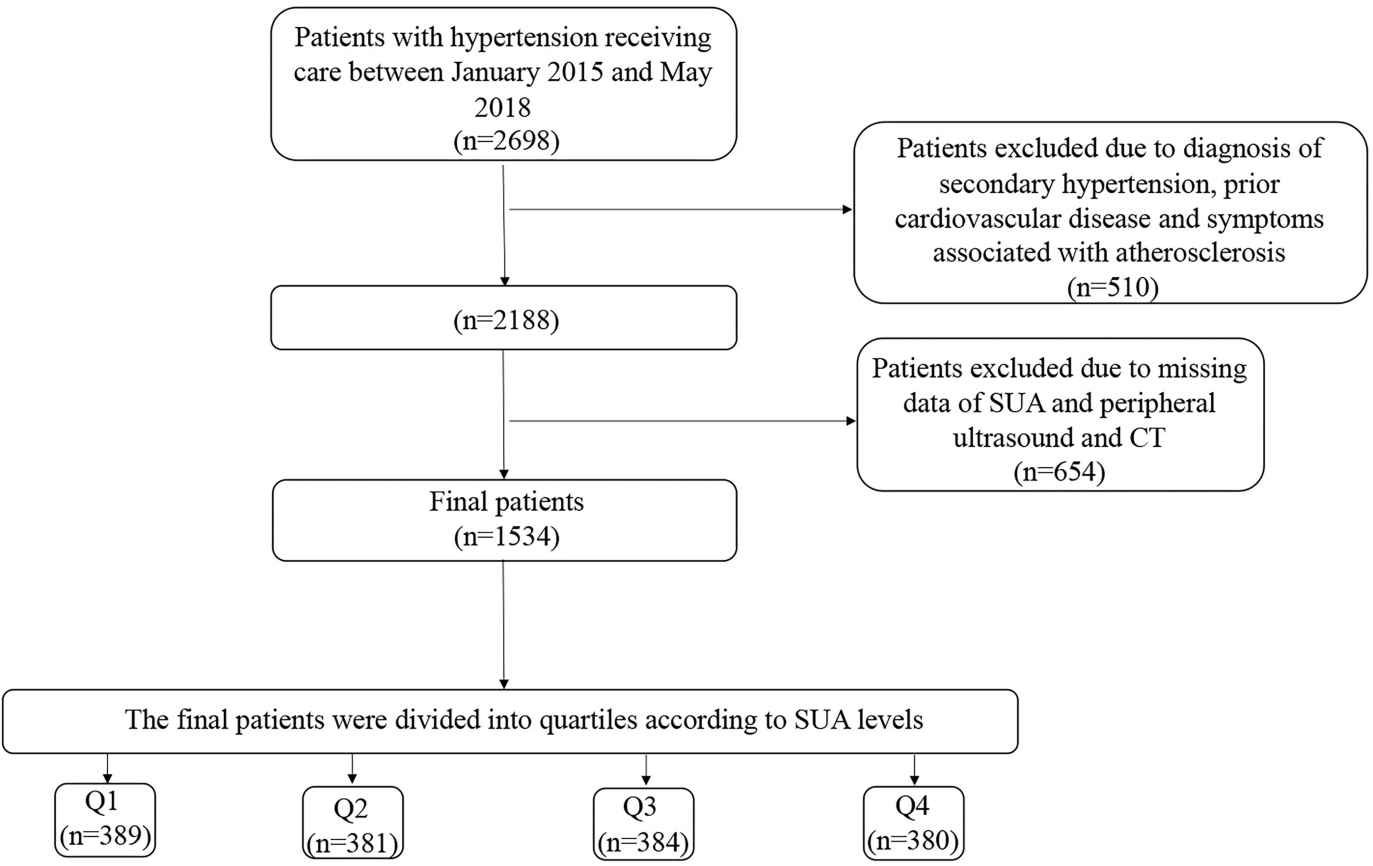
Flow Chart of the participants. The quartiles of SUA concentration were calculated separately for men and women. SUA, serum uric acid; CT, Computed tomography.

We examined hospitalized patients with hypertension, aged 18–80 years, between January 1, 2015 and May 31, 2018 at the First Affiliated Hospital of Dalian Medical University. Those with secondary hypertension, uncontrolled blood pressure, prior cardiovascular disease including coronary disease, stroke/transient ischemic attack, and those patients missing key clinical covariates were excluded. A total of 1,534 patients were included in the present study. A brief description of the study participants is given in Figure 1. The study was conducted under the guidelines of the Helsinki declaration. All protocols described here were performed under the approved guidelines. The study was approved by the First Affiliated Hospital of the Dalian Medical University Institutional Review Board, and the requirement for informed consent was waived.

### Definition of Explanatory Variables

Clinical characteristics, including age, sex, and major risk factors of hypertension (diabetes mellitus, arterial hypertension, dyslipidemia, smoking, family history of cardiovascular disease) were ascertained from electronic health records. Blood samples, regardless of fasting status, were collected, and all the biochemical parameters (glucose, triglyceride, low-density lipoprotein, total cholesterol, high-density lipoprotein, and creatinine levels) were performed at the First Affiliated Hospital of Dalian Medical University using the standard protocols. SUA levels were determined using the Uricase-Peroxidase method with an autoanalyzer (BECKMAN COULTER AU680 Chemistry Analyzer, USA). Diabetes mellitus was defined as fasting plasma glucose ≥126 mg/dL or treatment with insulin or oral hypoglycemic medication (13). Arterial hypertension was defined as systolic blood pressure ≥140 mmHg, diastolic blood pressure ≥90 mmHg, or current use of blood pressure-lowering medication (14). Dyslipidemia was defined as total cholesterol ≥240 mg/dL, low-density lipoprotein cholesterol ≥160 mg/dL, high density lipoprotein cholesterol <40 mg/dL, or use of lipid-lowering drugs (15, 16). Patients with a current history of smoking or reported a lifetime consumption of >100 cigarettes were defined as smokers (17, 18). The estimated glomerular filtration rate (eGFR) was calculated using the Modification of Diet in Renal Disease equation (19). Cardiovascular risk was evaluated by the 10-year risk of coronary heart disease of the Framingham Heart Study (FHS) (18, 20). FHS scores were classified as low (<10%), moderate (10–20%), or high (>20%) risk.

### Identification of SCA

Patients were examined by cervical vascular color ultrasonic inspection, coronary computer tomography (CT), renal artery CT, and thoracic aorta CT to evaluate SCA.

#### Vascular Ultrasound Imaging

We examined the bilateral carotid artery by using Color Doppler Ultrasonic diagnostic instrument (Philips iU-22 Ultrasound System, Philips Medical Systems, Bothell, WA, USA). Carotid intima-media thickness (IMT) was quantified by carotid ultrasound (21, 22). The presence of a plaque can be identified by an IMT ≥1.5 mm, or by a focal increase in thickness by more than 0.5 mm or 50% of the surrounding carotid IMT-value (23). The degree of carotid stenosis was graded according to the internal carotid artery peak systolic velocity (ICA PSV): (i) Normal: ICA PSV is <125 cm/s and no plaque or intimal thickening is visible; (ii) Minimal: 50% stenosis when ICA PSV is <125 cm/s and plaque or intimal thickening is visible; (iii) Mild: 50–69% stenosis when ICA PSV is 125–230 cm/s and plaque is visible; (iv) Moderate: 70% stenosis to near occlusion when ICA PSV is >230 cm/s and visible plaque and lumen narrowing are seen; (v) Severe: near occlusion when there is a markedly narrowed lumen at color Doppler US; and (vi) Occluded: total occlusion when there is no detectable patent lumen at the gray-scale US and no flow at spectral, power, and color Doppler US (24).

#### Computed Tomography Angiography

We evaluated the coronary arteries, thoracic aorta, and renal arteries using dual-source cardiovascular CT (SOMATOM Definition AS, Siemens Healthineers, Forchheim, Germany). The degree of stenosis was graded according to the inner diameter of the lumen: Normal: absence of plaque and no luminal stenosis; Minimal: plaque with <25% stenosis; Mild: 25–49% stenosis; Moderate: 50–69% stenosis; Severe: 70–99% stenosis; Occluded (25).

### Definition of Subclinical Atherosclerosis and Multi-Territorial Extent of Subclinical Atherosclerosis

SCA was defined as the availability of atherosclerotic plaques in the coronary, carotid, thoracic, and renal territories. The multi-territorial extent of SCA was defined according to the number of vascular sites affected. Patients were classified as disease-free (0 vascular sites affected), focal (1 site), intermediate (2 sites), or generalized (≥3 sites) SCA.

### Statistical Analysis

Data processing, management and statistical analysis were performed using SPSS (version 23). The population studied was stratified into quartiles based on gender-specific levels of SUA. The respective cut-off of SUA values for Q1, Q2, Q3, and Q4 were ≤ 356.00, 356.01–412.00, 412.01–467.00, and ≥467.00 μmol/L in men. The cut-off of SUA values for Q1, Q2, Q3, and Q4 in women were ≤320.00, 320.01–369.00, 369.01–433.00, and ≥433.01 μmol/L, respectively. Continuous and categorical variables were summarized using mean ± SD and percentiles, respectively. Analysis of variance was employed to compare three groups or more. The χ^2^-test was used to compare categorical variables. The likelihood of SCA associated with elevated SUA levels was calculated using a logistic regression model; SUA values were entered in the models as quartiles (with the first sex-specific quartile as the baseline reference) to assess the odds ratio (OR) and 95 percent confidence interval (95% CI). The logistic regression analysis was carried out in three models. Model 1 adjusted for age and sex. Model 2 adjusted for age, sex, eGFR, smoking, alcohol and diuretic use. Model 3 adjusted for age, sex, eGFR, smoking, alcohol intake, use of antihypertensive drugs, use of statins, blood pressure, diabetes and dyslipidemia. We further calculated OR and corresponding 95% CIs for the occurrence of SCA associated with 1 SD increase in SUA for patients who were classified according to FHS-10 year risk. All statistical analyses were two-sided, and a *P*-value of < 0.05 was considered statistically significant.

## Results

### Baseline Characteristics

This study included 1,534 patients (775 men and 759 women). The baseline characteristics of participants by SUA quartile (Q) are presented in Table 1. The values of the mean SUA, TG, creatinine and percentage of dyslipidemia were significantly higher in Q4 than in Q1–Q3. The values of the mean eGFR levels were significantly higher in Q1 than in Q2–Q4. The proportion of participants, who were in diuretic and statin use was higher in the highest quartile (Q4) compared with lower quartiles.

**Table 1:** Baseline characteristics of patients by serum uric acid (SUA) quartiles.

		**Quartiles of serum uric acid (*****n*** **=** **1,534)**				
**Variables**	**Total**	**Q1**	**Q2**	**Q3**	**Q4**	***P*-value**
Number of subjects	1,534	389	381	384	380	
SUA, μmol/L	397.70 ± 87.85	296.37 ± 38.86	366.34 ± 24.65	418.93 ± 26.26	511.40 ± 58.49	<0.001
Male, *N* (%)	775 (50.5)	196 (50.4)	193 (50.7)	193 (50.3)	193 (50.8)	0.999
Age, years	60.11 ± 13.33	60.70 ± 13.98	60.02 ± 13.36	60.63 ± 13.453	59.09 ± 12.47	0.307
Smoking, *N* (%)	440 (28.7)	108 (27.8)	98 (25.7)	117 (30.5)	117 (30.8)	0.518
Alcohol use, *N* (%)	362 (23.6)	87 (22.4)	82 (21.5)	95 (24.7)	98 (25.8)	0.798
SBP, mm Hg	130.45 ± 9.31	130.40 ± 9.45	129.74 ± 9.10	130.29 ± 9.69	131.33 ± 8.93	0.125
DBP, mm Hg	80.79 ± 9.11	80.02 ± 9.35	80.71 ± 8.96	81.51 ± 9.27	80.92 ± 8.79	0.153
Diabetes, *N* (%)	424 (27.6)	92 (23.7)	109 (28.6)	108 (28.1)	115 (30.3)	0.201
Dyslipidemia, *N* (%)	1,219 (79.7)	296 (76.7)	290 (76.3)	321 (83.6)	312 (82.1)	0.020
Statins use, *N* (%)	1,077 (70.2)	251 (64.5)	267 (70.1)	290 (75.5)	269 (70.8)	0.010
TC, mg/dL	193.17 ± 40.57	193.25 ± 40.64	193.47 ± 39.70	193.17 ± 41.66	192.80 ± 40.39	0.997
TG, mg/dL	163.20 ± 131.46	151.90 ± 146.58	146.6 ± 92.16	161.29 ± 113.66	193.28 ± 158.12	<0.001
HDL-c, mg/dL	47.65 ± 10.98	49.451 ± 11.43	47.806 ± 10.722	47.26 ± 10.89	46.04 ± 10.63	<0.001
LDL-c, mg/dL	107.88 ± 27.30	107.07 ± 26.94	108.59 ± 27.16	108.38 ± 27.80	107.49 ± 27.36	0.849
Creatinine, μmol//L	71.09 ± 41.59	66.69 ± 38.79	67.10 ± 18.35	71.87 ± 19.28	79.09 ± 41.59	<0.001
eGFR (mL/min/1.73 m^2^)	90.36 ± 21.00	94.87 ± 21.43	93.11 ± 18.91	88.51 ± 20.00	84.86 ± 22.07	<0.001
Diuretics use, *N* (%)	474 (30.9)	101 (26.0)	111 (29.1)	128 (33.3)	134 (35.3)	0.024
CCB use, *N* (%)	1,402 (91.4)	354 (91.0)	353 (92.7)	346 (90.1)	349 (91.8)	0.626
ACEI/ARB use, *N* (%)	501 (32.7)	116 (29.8)	101 (26.5)	138 (35.9)	146 (38.4)	0.001
β-blockers use, *N* (%)	758 (49.4)	174 (44.7)	183 (48.0)	192 (50.0)	209 (55.0)	0.037

*Data were presented as mean ± SD for continuous variable and number (percentage) for category variables. The quartiles of SUA concentration were calculated separately for men and women. The cut-off of SUA levels in men was ≤356.00, 356.01–412.00, 412.01–467.00 and ≥467.01 μmol/L. The cut-off of SUA levels in women was ≤ 320.00, 320.01–369.00, 369.01–433.00 and ≥433.01 μmol/L. SUA, serum uric acid; SBP, systolic blood pressure; DBP, diastolic blood pressure; TC total cholesterol; TG, triglyceride; HDL-C, high-density lipoprotein cholesterol; LDL-C, low-density lipoprotein cholesterol; eGFR, estimated glomerular filtration rate; CCB, Calcium channel blocker; ACEI, angiotensin-converting enzyme inhibitor; ARB, angiotensin II receptor blocker*.

### The Prevalence, Vascular Distribution, and Extent of Subclinical Atherosclerosis Across the Serum Uric Acid Levels

According to Table 2, plaques were discovered by ultrasound and CT angiography in 85.9% of the patients (51.6% in the carotids, 55.3% in the coronary, 74% in the thoracic aorta, and 44% in the renal arteries). Patients with asymptomatic thoracic aorta atherosclerosis accounted for the highest proportion (74%). Classification of participants according to the extent of atherosclerosis showed focal SCA in 17.9%, intermediate SCA in 21.3%, and generalized SCA in 46.6%. Among those participants having generalized SCA, the thoracic aorta was the most likely territory to be affected. With an increase of uric acid levels, the proportion of patients with SCA were increased. The prevalence of SCA increased from 78.9 to 92.1% across Q1–Q4 of SUA levels. Regardless of the territory of the artery, the degree of SCA was increased with an increase in uric acid concentration (*P* < 0.05).

**Table 2:** Prevalence of SCA according to quartiles of SUA levels.

	**Quartiles of SUA (*****N*** **=** **1,534)**					
	**Total**	**Q1 (*N* = 389)**	**Q2 (*N* = 381)**	**Q3 (*N* = 384)**	**Q4 (*N* = 380)**	***P-*value**
SUA, μmol/L	397.70 ± 87.85	296.37 ± 38.86	366.34 ± 24.65	418.93 ± 26.26	511.40 ± 58.49	<0.001
SCA, *N* (%)	1,317 (85.9)	307 (78.9)	318 (83.5)	342 (89.1)	350 (92.1)	<0.001
**Number of SCA sites**, ***N*** **(%)**						
0	217 (14.1)	82 (21.1)	83 (16.5)	42 (10.9)	30 (7.9)	<0.001
1	275 (17.9)	80 (20.6)	67 (17.6)	65 (16.9)	63 (16.6)	
2	327 (21.3)	87 (22.4)	67 (17.6)	89 (23.2)	84 (22.1)	
≥3	715 (46.6)	140 (36.0)	184 (48.3)	188 (49.0)	203 (53.4)	
**Distribution of SCA**, ***N*** **(%)**						
Carotid artery	792 (51.6)	157 (40.4)	202 (53.0)	215 (56.0)	218 (57.4)	<0.001
Coronary artery	848 (55.3)	158 (40.6)	208 (54.6)	215 (56.0)	267 (70.3)	<0.001
Thoracic aorta	1,135 (74.0)	273 (70.2)	272 (71.4)	290 (75.5)	300 (78.9)	0.022
Renal artery	702 (45.8)	147 (37.8)	174 (45.7)	199 (51.8)	182 (47.9)	0.001
**Severity of arterial stenosis**						
**Carotid artery**, ***N*** **(%)**						
Normal	743 (48.4)	232 (59.6)	179 (47.0)	169 (44.0)	163 (42.9)	<0.001
Minimal	763 (49.7)	152 (39.1)	198 (52.0)	207 (53.9)	206 (54.2)	
Mild-severe	28 (1.8)	5 (1.3)	4 (1.0)	8 (2.1)	11 (2.9)	
**Coronary artery**, ***N*** **(%)**						
Normal	686 (44.7)	231 (59.4)	173 (45.4)	169 (44.0)	113 (29.7)	<0.001
Minimal	539 (35.1)	117 (30.1)	145 (38.1)	126 (32.8)	151 (39.7)	
Mild	168 (11.0)	32 (8.2)	34 (8.9)	49 (12.8)	53 (13.9)	
Moderate-severe	141 (9.2)	9 (2.3)	29 (7.6)	40 (10.4)	63 (16.6)	
**Thoracic aorta**, ***N*** **(%)**						
Normal	399 (26.0)	116 (29.8)	109 (28.6)	94 (24.5)	80 (21.1)	0.043
Minimal	1,120 (73.0)	269 (69.2)	268 (70.3)	289 (75.3)	294 (77.4)	
Mild-severe	15 (1.0)	4 (1.0)	4 (1.0)	1 (0.3)	6 (1.6)	
**Renal artery**, ***N*** **(%)**						
Normal	832 (54.2)	242 (62.2)	207 (54.3)	185 (48.2)	198 (52.1)	0.003
Minimal	370 (24.1)	89 (22.9)	102 (26.8)	96 (25.0)	83 (21.8)	
Mild	248 (16.2)	47 (12.1)	56 (14.7)	74 (19.3)	71 (18.7)	
Moderate	46 (3.0)	5 (1.3)	10 (2.6)	16 (4.2)	15 (3.9)	
Severe	38 (2.5)	6 (1.5)	6 (1.6)	13 (3.4)	13 (3.4)	

*Data were presented as mean ± SD for continuous variable and number (percentage) for categorical variables. SUA, serum uric acid; SCA, subclinical atherosclerosis*.

### Elevated Serum Uric Acid Levels Increase the Risk of Subclinical Atherosclerosis

**Figure 2:**
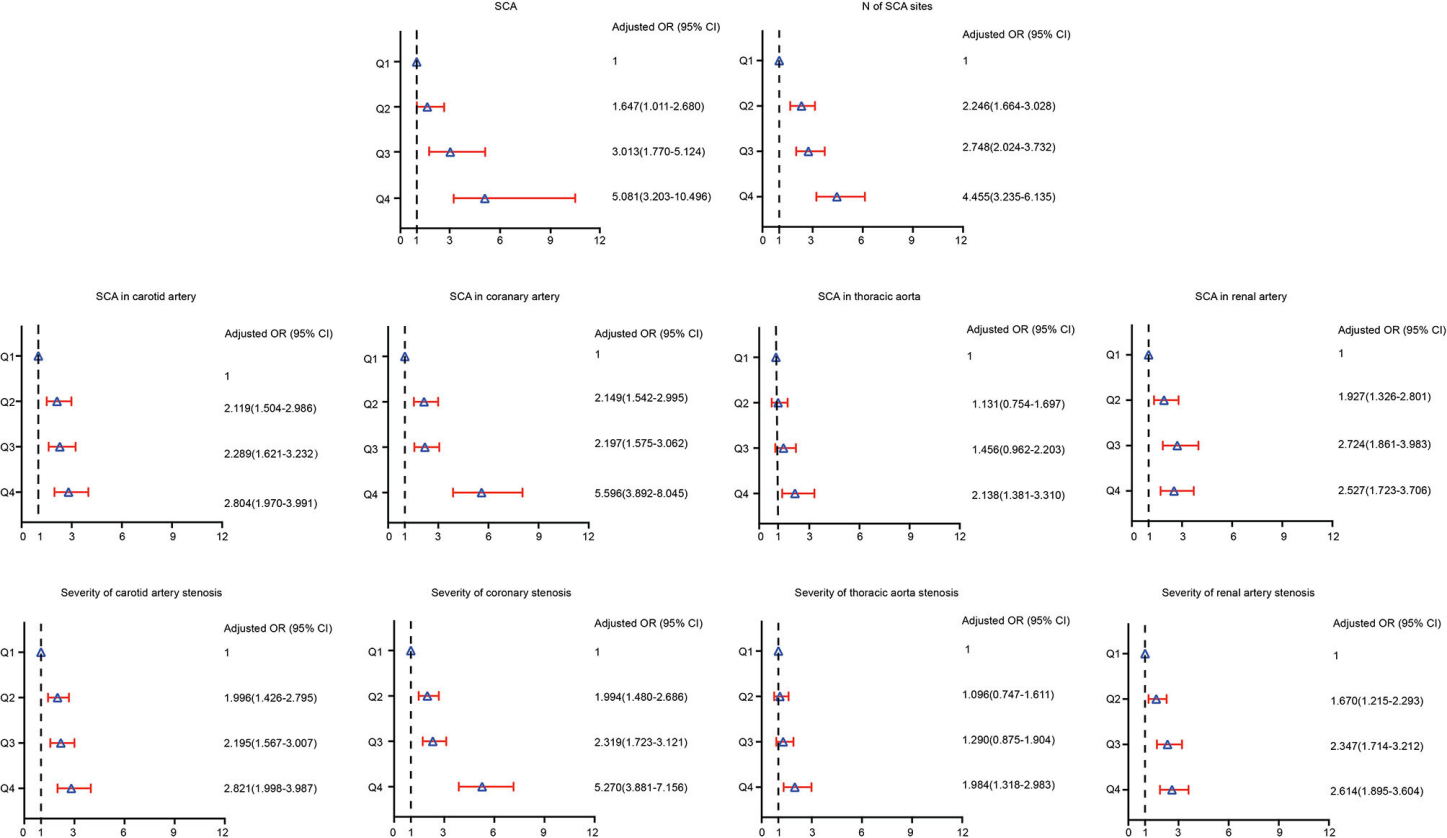
Risk of prevalence and severity of SCA according to baseline serum uric acid quartiles in adjusted models.

We used the logistic regression model to analyze the risk of SCA. The OR for SCA was analyzed in each SUA quartile, with the first quartile serving as the reference group. The association between SUA and the prevalence of SCA is presented in Table 3 and Supplementary Table 1. Compared with patients in the first quartile, the ORs (95% CI) for SCA in Q2, Q3, and Q4 were 1.647 (1.011–2.680), 3.013 (1.770–5.124) and 5.081 (3.203–10.496), respectively (*p* for trend <0.05). Also, patients with hypertension with elevated SUA levels had a higher likelihood of an increased number of SCA sites. The ORs (95% CI) for the number of SCA sites among patients with hypertension in Q2, Q3, and Q4 were 2.246 (1.664–3.028), 2.748 (2.024–3.732) and 4.455 (3.235–6.135), respectively (*p* for trend <0.05). As shown in Figure 2, patients in the highest SUA quartile had a significantly increased risk of SCA in the carotid artery, coronary artery, thoracic aorta, and renal artery even after adjusting for potential confounding factors, including age, sex, eGFR, smoking, alcohol use, antihypertensive drugs use, statins use, hypertension, diabetes, and dyslipidaemia.

**Table 3:** Risk of SCA according to baseline serum uric acid quartiles in adjusted models.

	**Quartiles of SUA (*****N*** **=** **1,534)**						
		**Q2 (*****N*** **=** **381)**		**Q3 (*****N*** **=** **384)**		**Q4 (*****N*** **=** **380)**	
	**Q1 (*N* = 389)**	**OR (95%CI)**	***P-*value**	**OR (95%CI)**	***P-*value**	**OR (95%CI)**	***P-*value**
SCA	1.000 (ref.)	1.647 (1.011–2.680)	0.045	3.013 (1.770–5.124)	<0.001	5.081 (3.203–10.496)	<0.001
Number of SUA sites	1.000 (ref.)	2.246 (1.664–3.028)	<0.001	2.748 (2.024–3.732)	<0.001	4.455 (3.235–6.135)	<0.001
SCA in carotid artery	1.000 (ref.)	2.119 (1.504–2.986)	<0.001	2.289 (1.621–3.232)	<0.001	2.804 (1.970–3.991)	<0.001
SCA in coronary artery	1.000 (ref.)	2.149 (1.542–2.995)	<0.001	2.197 (1.575–3.062)	<0.001	5.596 (3.892–8.045)	<0.001
SCA in thoracic aorta	1.000 (ref.)	1.131 (0.754–1.697)	0.552	1.456 (0.962–2.203)	0.076	2.138 (1.381–3.310)	0.001
SCA in renal artery	1.000 (ref.)	1.927 (1.326–2.801)	0.001	2.724 (1.861–3.983)	<0.001	2.527 (1.723–3.706)	<0.001
Severity of carotid artery stenosis	1.000 (ref.)	1.996 (1.426–2.795)	<0.001	2.195 (1.567–3.077)	<0.001	2.821 (1.998–3.987)	<0.001
Severity of coronary stenosis	1.000 (ref.)	1.994 (1.480–2.686)	<0.001	2.319 (1.723–3.121)	<0.001	5.270 (3.881–7.156)	<0.001
Severity of thoracic aorta stenosis	1.000 (ref.)	1.096 (0.747–1.611)	0.638	1.290 (0.875–1.904)	0.199	1.984 (1.318–2.983)	0.001
Severity of renal artery stenosis	1.000 (ref.)	1.670 (1.215–2.293)	0.002	2.347 (1.714–3.212)	<0.001	2.614 (1.895–3.604)	<0.001

*Adjusted for age, sex, eGFR, smoking, alcohol use, antihypertensive drugs use, statins use, blood pressure, diabetes, and dyslipidaemia. SUA, serum uric acid; SCA, subclinical atherosclerosis; OR, odds ratio; CI, Confidence Interval*.

Similarly, patients in the highest quartile of SUA had a higher risk of severe SCA. For instance, the ORs (95% CI) for carotid atherosclerosis in those patients who were in Q2, Q3, and Q4 were 1.996 (1.426–2.795), 2.195 (1.567–3.077), and 2.821 (1.998–3.987), respectively (*p* for trend <0.05). Patients in the higher quartiles were more likely to have severe coronary, thoracic and renal SCA than those in the first quartile. Compared with patients in the first quartile, the ORs (95% CI) for coronary, thoracic, and renal SCA in Q4 were 5.270 (3.881–7.156), 1.984 (1.318–2.983), and 2.614 (1.895–3.604), respectively (*P* for trend across quartiles for all SCA was <0.05).

### Elevated Serum Uric Acid Levels Associated With the Risk of Subclinical Atherosclerosis in All Categories of FHS 10-Year Risk

**Figure 3:**
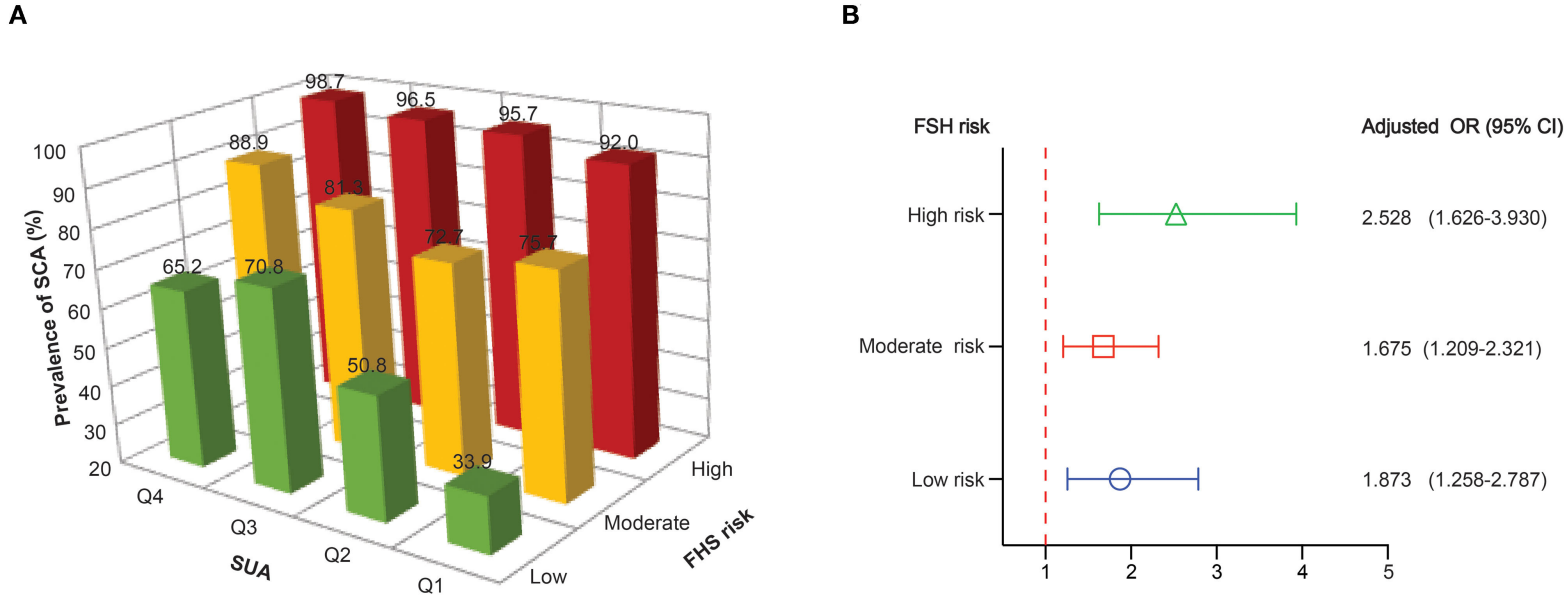
**(A)** The prevalence of subclinical atherosclerosis based on SUA quartiles in patients grouped by FHS-10 year risk. **(B)** Adjusted odds ratios for subclinical atherosclerosis for 1 SD increase in SUA in patients grouped by FHS-10 year risk.

Although the FHS 10-year risk was designed to assess the risk of cardiovascular events derived from atherosclerosis, not the presence of SCA, we aimed to complement these predictive models by comparing the presence and extent of subclinical disease across different risk categories. Most patients with an increased cardiovascular risk scale, 10-year FHS risk, had a higher prevalence of SCA (Supplementary Table 2). Patients classified at high 10-year FHS risk had the highest prevalence of SCA (95.8%), and 84% of the high-risk patients had SCA in two or more territories (84.2%). Among patients at moderate 10-year FHS risk, 79.8% had SCA. Also, extensive SCA was present in a substantial number of patients among the low-risk category (53.8%). Figure 3A describes the prevalence of SCA based on SUA quartiles in patients grouped by FHS-10 year risk. Compared to the patients at the lower quartiles of SUA, those patients at higher quartiles of SUA levels had a higher proportion of SCA, with high FHS-10 year risk patients in Q4 accounts for the highest risk of SCA (98.7%). However, substantial SCA risk was present among those patients in the highest quartile of SUA levels classified at the low-risk category. Table 4 describes the adjusted odds ratios for SCA according to baseline serum uric acid quartile in patients grouped by FHS-10 year risk. The OR (CI) for SCA for patients in quartile four classified at low, moderate and high FSH risk were 4.036 (1.564, 10.416), 4.262 (1.689, 10.754), and 10.889 (3.034, 39.078), respectively. Also, an increase in one standard deviation of SUA (87.85 μmol/L) was significantly associated with SCA in those patients regardless of the different category of FHS 10-year risk (Figure 3B), with the ORs for SCA at low, moderate and high FHS risk groups were 1.873, 1.675, and 2.528, respectively.

**Table 4:** Risk of SCA according to baseline serum uric acid quartile in adjusted models in patients grouped by FHS-10 year risk.

	**Quartiles of SUA (*****N*** **=** **1,534)**						
	**Q1 (*N* = 389)**	**Q2 (*****N*** **=** **381)**		**Q3 (*****N*** **=** **384)**		**Q4 (*****N*** **=** **380)**	
		**OR (95%CI)**	***P-*value**	**OR (95%CI)**	***P-*value**	**OR (95%CI)**	***P-*value**
Low	1.000 (ref.)	2.271 (0.938, 5.496)	0.069	5.439 (2.025, 14.608)	0.001	4.036 (1.564, 10.416)	0.004
Moderate	1.000 (ref.)	0.795 (0.362, 1.748)	0.568	1.368 (0.616, 3.039)	0.442	4.262 (1.689, 10.754)	0.002
High	1.000 (ref.)	2.402 (1.032, 5.593)	0.042	3.602 (1.431, 9.07)	0.007	10.889 (3.034, 39.078)	<0.001

*OR, odds ratio; CI, Confidence Interval*.

## Discussion

Using a multi-territorial evaluation, the present study discovers a high prevalence of SCA (85.9%) in patients with hypertension, with nearly half of patients classified as having generalized SCA (46.6%). Also, the findings of this cross-sectional analysis clarified the relationship between elevated SUA and the presence of SCA in the Chinese population with hypertension. Those participants who had a high concentration of SUA were more likely to have SCA. Even after adjustment for possible confounders, the association between elevated SUA concentrations and SCA remained significant. Similarly, most individuals classified at high risk by traditional scale (10-year FHS risk) had a higher prevalence, vascular distribution, and multi-territorial extent of SCA. However, extensive SCA was also present in a substantial number of low-risk patients at the higher quartiles of SUA classified at low FHS-10 year risk, suggesting elevated SUA associates with SCA risk to some extent regardless of FHS risk.

Uric acid is the final product of purine metabolism (26), mainly produced in the liver and gastrointestinal tract and excreted by the kidney (27). Epidemiological studies reported positive associations in blood pressure readings with an increase in SUA (28), whereas some documented evidence reported there exists a significant association between SUA and hypertension (29). Hypertension and plasma aldosterone concentration have been proved to be strongly associated with surrogate markers of SCA (30, 31) whereas other potential risk factors for SCA were rarely reported. In the present study, the proportion of patients with SCA were increased with an increase in SUA levels. Participants in the higher quartiles of SUA levels had a higher likelihood of having SCA. Importantly, the degree of atherosclerosis was found to increase with an increase in SUA levels. Atherosclerosis is the leading cause of cardiovascular death and disabilities such as sudden death, myocardial infarction, or stroke (32). Therefore, it is of great significance to take effective measures to intervene before individuals develop into the clinical phase or end-stage.

According to our present study, the thoracic aorta is the most frequently affected vascular site. However, the previous study reported a strong prevalence of SCA in the iliofemoral arteries (82%) among the low-risk population (3). In the present study, the iliofemoral region has not been studied therefore the comparison between the thoracic aorta and iliofemoral arteries cannot be done. Overall, among the 1,534 patients with hypertension included in the present study, 85.9% of patients were presented with SCA, which indicates a higher prevalence of subclinical atherosclerosis than the prevalence of SCA (63%) reported in the PESA study among the general population (3). These differences in the prevalence of SCA is probably attributable to (i) the examination of different territories, which were not explored in earlier studies (for instance thoracic aorta), (ii) investigation of more territories, and (iii) examination of hypertension individuals who are already at higher risk of SCA. Given the high prevalence of SCA in the thoracic aorta (74%), SCA assessment tends to be more important. Thus, imaging of thoracic aorta may be a useful screening tool among the hypertension population for detecting atherosclerosis in its early stages.

Both the Asymptomatic Polyvascular Abnormalities Community study (APACS) (33) and the Brisighella Heart Study (34) reported a positive correlation between an increase in hyperuricemia rates and the risk of atherosclerosis. In the present study, the patients with hypertension in the higher quartiles of SUA had lower eGFR. Thus, its negative implication in compromising the predictive power of SUA may not be underestimated. Nevertheless, the present study indicated that patients with hypertension in the top quartile of SUA had an increased risk of SCA, even after adjusting for conventional risk factors for CVD, including the body mass index (BMI), eGFR, and dyslipidemia. Regardless of the FHS-10 year risk category, those patients at higher quartile of SUA levels had a higher OR for SCA. These findings suggest that individuals with high SUA levels, regardless of the status of their FHS 10-year risk, are remained at risk of SCA. Notably, the patients with low FHS-10 year risk in the Q3 (OR = 5.439), and Q4 (OR = 4.036) showed a tremendous risk of SCA, indicating the strong relationship between SUA level and SCA. Therefore, community health strategies aimed at SCA prevention among hypertension individuals, whose SUA levels and FHS 10 years risk score increased greater than the standard cut-off points should be accompanied by ultrasound or CT evaluations to improve the treatment outcomes and avoid serious complications of SCA.

This research connected elevated SUA with asymptomatic pre-clinical arterial atherosclerosis. The result of these findings gives novel insights into the overall distribution of SCA among the Chinese population with hypertension. Future studies are needed to investigate whether SUA modulation could delay or even prevent SCA. To our knowledge, this is the first study to investigate the relationship between the SUA and asymptomatic atherosclerosis rates in a Chinese population with hypertension. In our study, 79.7% of the population had dyslipidemia and 70% had statin use, which may mitigate the bias associated with the confounding effect of dyslipidemia. However, there are some drawbacks. Firstly, due to the limitations of cross-sectional nature, the cause-and-effect relationship between SUA and SCA was not discussed. Second, the present study does not include evidence on the underlying cause of stenosis, which makes the root of arterial stenosis difficult to categorize. Therefore, we propose an additional longitudinal study to provide epidemiological evidence for the relationship between elevated SUA and incident SCA among the hypertension population in China.

## Data Availability Statement

The raw data supporting the conclusions of this article will be made available by the authors, without undue reservation.

## Author Contributions

YX and XY designed the study. FL and SH contributed in the study protocol, literature searches, data collection, and statistical analysis and were involved in the final draft of the manuscript. TH, YJ, YZ, YL, HL, and SL contributed in the coordination of designing and analysis. All authors have read and approved the final manuscript.

## Conflict of Interest

The authors declare that the research was conducted in the absence of any commercial or financial relationships that could be construed as a potential conflict of interest.
