# Supplementary material for: The Prevalence, Distribution, and Extent of Subclinical Atherosclerosis and Its Relation With Serum Uric Acid in Hypertension Population
Source: Front Cardiovasc Med. 2021 Apr 15;8:638992. doi: 10.3389/fcvm.2021.638992 (PMC8081824; doi:10.3389/fcvm.2021.638992)
Supplement: Supplementary file 1 [file Data_Sheet_1.docx]

**Table S1: Risk of SCA according to baseline serum uric acid quartiles in adjusted models**

|  | **Quartiles of SUA（N=1534）** | | | | | | | | | |
| --- | --- | --- | --- | --- | --- | --- | --- | --- | --- | --- |
|  | Q1（N=389） |  | Q2（N=381） | |  | Q3（N=384） | |  | Q4（N=380） | |
|  |  |  | OR (95%CI) | P trend |  | OR (95%CI) | P trend |  | OR (95%CI) | P trend |
| **SCA** | | | | | | | | | | |
| Model 1 | 1.000(ref.) |  | 1.690(1.053-2.714) | 0.03 |  | 3.053(1.836-5.077) | <0.001 |  | 5.766(3.307-10.061) | <0.001 |
| Model 2 | 1.000(ref.) |  | 1.747(1.084-2.815) | 0.022 |  | 3.124(1.857-5.249) | <0.001 |  | 6.147(3.421-11.045) | <0.001 |
| Model 3 | 1.000(ref.) |  | 1.647(1.011-2.680) | 0.045 |  | 3.013(1.770-5.124) | <0.001 |  | 5.081(3.203-10.496) | <0.001 |
| **Number of SUA sites** | | | | | | | | | | |
| Model 1 | 1.000(ref.) |  | 2.243(1.675-3.007) | <0.001 |  | 2.835(2.109-3.815) | <0.001 |  | 4.500(3.320-6.098) | <0.001 |
| Model 2 | 1.000(ref.) |  | 2.342(1.744-3.146) | <0.001 |  | 2.878(2.130-3.892) | <0.001 |  | 4.623(3.370-6.334) | <0.001 |
| Model 3 | 1.000(ref.) |  | 2.246(1.664-3.028) | <0.001 |  | 2.748(2.024-3.732) | <0.001 |  | 4.455(3.235-6.135) | <0.001 |
| **SCA in Carotid artery** | | | | | | | | | | |
| Model 1 | 1.000(ref.) |  | 2.153(1.545-3.001) | <0.001 |  | 2.452(-1.758-3.421) | <0.001 |  | 2.968(2.125-4.150) | <0.001 |
| Model 2 | 1.000(ref.) |  | 2.212(1.582-3.096) | <0.001 |  | 2.440(1.740-3.421) | <0.001 |  | 2.939(2.079-4.154) | <0.001 |
| Model 3 | 1.000(ref.) |  | 2.119(1.504-2.986) | <0.001 |  | 2.289(1.621-3.232） | <0.001 |  | 2.804(1.970-3.991) | <0.001 |
| **SCA in Coronary artery** | | | | | | | | | | |
| Model 1 | 1.000(ref.) |  | 2.121(1.540-2.9180 | <0.001 |  | 2.214(1.610-3.050) | <0.001 |  | 5.150(3.673-7.221) | <0.001 |
| Model 2 | 1.000(ref.) |  | 2.186(1.584-3.013) | <0.001 |  | 2.303(1.665-3.1870) | <0.001 |  | 5.624(3.955-8.004) | <0.001 |
| Model 3 | 1.000(ref.) |  | 2.149(1.542-2.995) | <0.001 |  | 2.197(1.575-3.062) | <0.001 |  | 5.596(3.892-8.045) | <0.001 |
| **SCA in Thoracic aorta** | | | | | | | | | | |
| Model 1 | 1.000(ref.) |  | 1.169(0.784-1.742) | 0.443 |  | 1.505(1.004-2.255) | 0.048 |  | 2.284(1.505-3.466) | <0.001 |
| Model 2 | 1.000(ref.) |  | 1.169(0.782-1.747) | 0.447 |  | 1.451(0.963-2.184) | 0.075 |  | 2.153(1.399-3.313) | <0.001 |
| Model 3 | 1.000(ref.) |  | 1.131(0.754-1.697) | 0.552 |  | 1.456(0.962-2.203) | 0.076 |  | 2.138(1.381-3.310) | 0.001 |
| **SCA in Renal artery** | | | | | | | | | | |
| Model 1 | 1.000(ref.) |  | 1.972(1.374-2.832) | <0.001 |  | 2.807(1.946-4.043) | <0.001 |  | 2.643(1.840-3.796) | <0.001 |
| Model 2 | 1.000(ref.) |  | 1.986(1.379-2.858) | <0.001 |  | 2.784(1.921-4.035) | <0.001 |  | 2.675(1.837-3.892) | <0.001 |
| Model 3 | 1.000(ref.) |  | 1.927(1.326-2.801) | 0.001 |  | 2.724(1.861-3.983) | <0.001 |  | 2.527(1.723-3.706) | <0.001 |
| **Severity of Carotid artery stenosis** | | | | | | | | | | |
| Model 1 | 1.000(ref.) |  | 2.048(1.478-2.841) | <0.001 |  | 2.354(1.701-3.261) | <0.001 |  | 3.001(2.162-4.170) | <0.001 |
| Model 2 | 1.000(ref.) |  | 2.085(1.501-2.895) | <0.001 |  | 2.340(1.682-3.254) | <0.001 |  | 2.959(2.111-4.154) | <0.001 |
| Model 3 | 1.000(ref.) |  | 1.996(1.426-2.795) | <0.001 |  | 2.195(1.567-3.077) | <0.001 |  | 2.821(1.998-3.987) | <0.001 |
| **Severity of Coronary stenosis** | | | | | | | | | | |
| Model 1 | 1.000(ref.) |  | 2.020(1.513-2.697) | <0.001 |  | 2.349(-1.763-3.130) | <0.001 |  | 5.008(3.743-6.693) | <0.001 |
| Model 2 | 1.000(ref.) |  | 2.044(1.530-2.732) | <0.001 |  | 2.416(1.806-3.232) | <0.001 |  | 5.317(3.943-7.178) | <0.001 |
| Model 3 | 1.000(ref.) |  | 1.994(1.480-2.686) | <0.001 |  | 2.319(1.723-3.121) | <0.001 |  | 5.270(3.881-7.156) | <0.001 |
| **Severity of Thoracic aorta stenosis** | | | | | | | | | | |
| Model 1 | 1.000(ref.) |  | 1.142(0.783-1.667) | 0.49 |  | 1.368(0.936-1.998) | 0.106 |  | 2.181(1.476-3.225) | <0.001 |
| Model 2 | 1.000(ref.) |  | 1.135(0.776-1.662) | 0.511 |  | 1.307(0.890-1.919) | 0.172 |  | 2.024(1.350-3.034) | 0.001 |
| Model 3 | 1.000(ref.) |  | 1.096(0.747-1.611) | 0.638 |  | 1.290(0.875-1.904) | 0.199 |  | 1.984(1.318-2.983) | 0.001 |
| **Severity of Renal artery stenosis** | | | | | | | | | | |
| Model 1 | 1.000(ref.) |  | 1.751(1.281-2.389) | <0.001 |  | 2.479(1.824-3.370) | <0.001 |  | 2.872(2.106-3.912) | <0.001 |
| Model 2 | 1.000(ref.) |  | 1.742(1.274-2.380) | 0.001 |  | 2.418(1.772-3.297) | <0.001 |  | 2.804(2.038-3.854) | <0.001 |
| Model 3 | 1.000(ref.) |  | 1.670(1.215-2.293) | 0.002 |  | 2.347(1.714-3.212) | <0.001 |  | 2.614(1.895-3.604) | <0.001 |

Model 1: adjusted for age and sex.

Model 2: adjusted for age, sex, eGFR,smoking, alcohol use and diuretics use.

Model 3: adjusted for age, sex, eGFR,smoking, alcohol use, diuretics use, statins use, blood pressure, diabetes and dyslipidaemia.

**Table S2：Prevalence of SCA** **according to 10-year FHS risk**

|  | **Low FHS risk (N=212)** | | | | |
| --- | --- | --- | --- | --- | --- |
|  | **Total** | **Q1（n=59）** | **Q2（n=59）** | **Q3（n=48）** | **Q4 (n=46）** |
| SUA, μmol/L | 385.38±94.866 | 287.42±41.730 | 357.59±19.921 | 411.56±25.086 | 519.36±78.172 |
| SCA, N (%) | 114（53.8） | 20（33.9） | 30（50.8） | 34（70.8） | 30（65.2） |
| Number of SCA sites |  |  |  |  |  |
| 0, N (%) | 98（46.2） | 39（66.1） | 29（49.2） | 14（29.2） | 16（34.8） |
| 1, N (%) | 63（29.7） | 12（20.3） | 17（28.8） | 20（41.7） | 14（30.4） |
| 2, N (%) | 37（17.5） | 5（8.5） | 10（16.9） | 12（25.0） | 10（21.7） |
| ≥3, N (%) | 14（6.6） | 3（5.1） | 3（5.1） | 2（4.2） | 6（13.0） |
| Distribution of SCA, N (%) |  |  |  |  |  |
| Carotid artery | 34（16.0） | 5（8.5） | 13（22.0） | 9（18.8） | 7（15.2） |
| Coronary artery | 43（20.3） | 9（15.3） | 9（15.3） | 10（20.8） | 15（32.6） |
| Thoracic aorta | 81（38.2） | 13（22.0） | 20（33.9） | 23（47.9） | 25（54.3） |
| Renal artery | 24（11.3） | 4（6.8） | 5（8.5） | 8（16.7） | 7（15.2） |
|  | **Moderate FHS risk (N=397)** | | | | |
|  | **Total** | **Q1（n=103）** | **Q2（n=88）** | **Q3（n=107）** | **Q4（n=99）** |
| SUA, μmol/L | 394.75±88.497 | 294.91±38.981 | 359.28±25.545 | 414.06±26.791 | 509.30±58.655 |
| SCA, N (%) | 317（79.8） | 78（75.7） | 64（72.7） | 87（81.3） | 88（88.9） |
| Number of SCA sites |  |  |  |  |  |
| 0, N (%) | 80（20.2） | 25（24.3） | 24（27.3） | 20（18.7） | 11（11.1） |
| 1, N (%) | 105（26.4） | 31（30.1） | 20（22.7） | 23（21.5） | 31（31.3） |
| 2, N (%) | 111（28.0） | 27（26.2） | 19（21.6） | 37（34.6） | 28（28.3） |
| ≥3, N (%) | 101（25.4） | 20（19.4） | 25（28.4） | 27（25.2） | 29（29.3） |
| Distribution of SCA, N (%) |  |  |  |  |  |
| Carotid artery | 146（36.8） | 31（30.1） | 30（34.1） | 41（38.3） | 44（44.4） |
| Coronary artery | 153（55.3） | 30（29.1） | 31（35.2） | 40（37.4） | 52（52.5） |
| Thoracic aorta | 257（64.7） | 65（63.1） | 53（60.2） | 74（69.2） | 65（65.7） |
| Renal artery | 114（28.7） | 23（22.3） | 28（31.8） | 36（33.6） | 27（27.3） |
|  | **High FHS risk (N=923)** | | | | |
|  | **Total** | **Q1（n=226）** | **Q2 (n=233）** | **Q3（229）** | **Q4（n=235）** |
| SUA, μmol/L | 401.89±85.697 | 299.33±37.877 | 371.12±24.234 | 422.75±25.644 | 510.73±53.953 |
| SCA, N (%) | 884（95.8） | 208（92.0） | 223（95.7） | 221（96.5） | 232（98.7） |
| Number of SCA sites |  |  |  |  |  |
| 0, N (%) | 39（4.2） | 18（8.0） | 10（4.3） | 8（3.5） | 3（1.3） |
| 1, N (%) | 107（11.6） | 37（16.4） | 30（12.9） | 22（9.6） | 18（7.7） |
| 2, N (%) | 178（19.3） | 54（23.9） | 38（16.3） | 40（17.5） | 46（19.6） |
| ≥3, N (%) | 599（64.9） | 117（36.0） | 155（48.3） | 159（49.0） | 168（53.4） |
| Distribution of SCA, N (%) |  |  |  |  |  |
| Carotid artery | 611（66.2） | 121（53.5） | 158（67.8） | 165（72.1） | 167（71.1） |
| Coronary artery | 651（70.5） | 119（52.7） | 167（71.7） | 165（72.1） | 200（85.1） |
| Thoracic aorta | 795（86.1） | 194（85.8） | 198（85.0） | 193（84.3） | 210（89.4） |
| Renal artery | 562（60.9） | 119（52.7） | 140（60.1） | 155（67.7） | 148（63.0） |

Data were presented as mean ± SD for continuous variable and number (percentage) for categorical variables. SUA, serum uric acid; SCA, subclinical atherosclerosis;
